# Antiglycation and Antioxidant Properties of *Momordica charantia*

**DOI:** 10.1371/journal.pone.0159985

**Published:** 2016-08-11

**Authors:** Ali Aljohi, Sabine Matou-Nasri, Nessar Ahmed

**Affiliations:** 1 School of Healthcare Science, Manchester Metropolitan University, Manchester, United Kingdom; 2 King Abdullah International Medical Research Center, King Saud Bin Abdulaziz University for Health Sciences, Medical Genomics Research Department, Ministry of National Guard Health Affairs, Riyadh, Saudi Arabia; Consiglio Nazionale delle Ricerche, ITALY

## Abstract

The accumulation of advanced glycation endproducts (AGEs) and oxidative stress underlie the pathogenesis of diabetic complications. In many developing countries, diabetes treatment is unaffordable, and plants such as bitter gourd (or bitter melon; *Momordica charantia*) are used as traditional remedies because they exhibit hypoglycaemic properties. This study compared the antiglycation and antioxidant properties of aqueous extracts of *M*. *charantia* pulp (MCP), flesh (MCF) and charantin *in vitro*. Lysozyme was mixed with methylglyoxal and 0–15 mg/ml of *M*. *charantia* extracts in a pH 7.4 buffer and incubated at 37°C for 3 days. Crosslinked AGEs were assessed using gel electrophoresis, and the carboxymethyllysine (CML) content was analyzed by enzyme-linked immunosorbent assays. The antioxidant activities of the extracts were evaluated using assays to assess DPPH (1,1-diphenyl-2-picryl-hydrazyl) and hydroxyl radical scavenging activities, metal-chelating activity and reducing power of the extracts. The phenolic, flavonol and flavonoid content of the extracts were also determined. All extracts inhibited the formation of crosslinked AGEs and CML in a dose-dependent manner, with MCF being the most potent. The antioxidant activity of MCF was higher than that of MCP, but MCP showed the highest metal-chelating activity. MCF had the highest phenolic and flavonoid contents, whereas MCP had the highest flavonol content. *M*. *charantia* has hypoglycaemic effects, but this study shows that *M*. *charantia* extracts are also capable of preventing AGE formation *in vitro*. This activity may be due to the antioxidant properties, particularly the total phenolic content of the extracts. Thus, the use of *M*. *charantia* deserves more attention, as it may not only reduce hyperglycaemia but also protect against the build-up of tissue AGEs and reduce oxidative stress in patients with diabetes.

## Introduction

Diabetes mellitus is characterized by hyperglycaemia, and affected patients are prone to long-term complications [1, 2]. Hyperglycaemia plays a crucial role in the development of these complications via increased protein glycation. Protein glycation occurs via a nucleophilic addition reaction between a carbonyl group from a reducing sugar and a free protein amino group, resulting in the formation of freely reversible Schiff bases, which are rapidly rearranged to form more stable Amadori products. In the presence of transition metals and oxygen, glucose and Amadori products undergo autoxidation (autoxidative glycation and glycoxidation, respectively) to form free radicals [3, 4]. The free radicals then cause damage to biomolecules in the body [5].

AGEs may react with cellular receptors for AGEs to produce oxidative stress and proinflammatory molecules. AGE accumulation in tissues and oxidative stress underlies the long-term complications of diabetes mellitus [6]. In developing countries, conventional diabetes treatments are expensive, and traditional plant remedies are often used. The use of several traditional plant-based preparations, including those from *Momordica charantia* (also known as bitter gourd), as oral hypoglycaemic agents has been the subject of scientific evaluation [7]. *M*. *charantia* has been used as a medicinal plant for the management of hypertension and diabetes. Several studies have reported the antidiabetic and antilipidemic properties of *M*. *charantia* [8, 9].

The effect of *M*. *charantia* on AGE formation is unknown and deserves attention, as plant-based preparations could potentially be used as cost effective, non-toxic supplements with antiglycation properties to prevent or delay the onset of diabetic complications. In some communities, the outer green part of *M*. *charantia* (*M*. *charantia* flesh, MCF) is normally used for cooking, whereas in others, the inside of the vegetable (*M*. *charantia* pulp, MCP) is used. Charantin, a key constituent of *M*. *charantia*, is believed to have potential antidiabetic properties [10].

In the current study, we evaluated and compared the antioxidant properties of MCP, MCF and charantin *in vitro*, as well as their antiglycation properties in terms of inhibiting the formation of crosslinked AGEs and oxidative AGEs, such as carboxymethyllysine (CML).

## Materials and Methods

### Materials

All reagents including lysozyme, methylglyoxal, bovine serum albumin (BSA), EDTA, 1,1-diphenyl-2-picryl-hydrazyl (DPPH), Folin-Ciocalteu reagent, ferrous sulfate (FeSO_4_), hydrogen peroxide and sodium salicylate were obtained from Sigma-Aldrich (Dorset, UK) unless mentioned otherwise. Potassium ferricyanide (K_3_Fe(CN)_6_) was purchased from Fluka Chemicals Ltd (Gillingham, UK). The OxiSelect N-(carboxymethyl) lysine (CML) kit was purchased from Cell Biolabs (San Diego, CA, USA). Fresh *M*. *charantia* was purchased from a local Asian food store (Manchester, UK). Charantin was obtained from Shaanxi Honghao Bio-Tech (Shaanxi Province, China).

### Preparation of extracts

Aqueous flesh and pulp extracts were prepared according to a method described previously [11], with some modifications. The flesh and pulp (130 mg of each) of *M*. *charantia* were extracted using 1.3 ml of methanol. The samples were homogenized in a kitchen blender at the highest speed setting in 1 minute bursts, for a total burst time of 12 minutes. The homogenized extract was filtered through cheesecloth. A rotary evaporator was used to evaporate the methanol, and any remaining methanol was evaporated in a 100°C water bath.

### Preparation of AGEs

Lysozyme (10 mg/ml) was reacted with 0.1 M methylglyoxal and 0–15 mg/ml MCP, MCF or charantin in 0.1 M sodium phosphate buffer containing 3 mM sodium azide at pH 7.4, and then incubated at 37°C for 3 days, after which the samples were removed and stored at −20°C until analysis [12].

#### Preparation of CML

CML-modified proteins were prepared using a previously established method [13]. BSA (100 mg) was incubated with 3 mg of glyoxylic acid and 10 mg of NaCNBH_3_ in 10 ml of 0.2 M sodium phosphate buffer (pH 7.8) at 37°C for 24 hours, after which the sample was dialyzed against distilled water. Different concentrations (5–15 mg/ml) of the MCP, MCF and charantin extracts were included in the incubation mixture, depending on the treatment. After incubation, aliquots were removed and stored at −20°C until further analysis.

### Measurement of crosslinked AGEs by SDS-PAGE

Crosslinked AGEs were assessed using sodium dodecyl sulfate-polyacrylamide gel electrophoresis (SDS-PAGE), in which 10 μg of protein was applied to each well, followed by electrophoresis and Coomassie blue staining of the gels [14]. The gels were photographed using the GeneSnap program from G Box Chem HR16 Bioimaging system (Syngene, Cambridge, UK). Bands within the same gel were compared. Integrated density (ID) was measured to analyze the one-dimensional electrophoretic gels, and the percentage of crosslinked AGEs was calculated using the following formula:

Formation of crosslinked AGEs (%)=(ID with inhibitor|ID without inhibitor)×100

### Measurement of CML using enzyme-linked immunosorbent assays (ELISA)

The samples (100 μl) were transferred to 96-well plates and incubated overnight at 4°C; next, the wells were rinsed twice with 250 μl of phosphate-buffered saline. The plates were blocked with 200 μl of blocking solution for 2 hours at room temperature with shaking and then washed three times using 1× washing buffer. Diluted anti-CML primary antibody (100 μl) was added to the wells, and the plates were then incubated at room temperature for 1 hour with shaking; all wells were subsequently washed three times using 1× washing buffer. A diluted HRP-conjugated secondary antibody (100 μl) was added to the wells, and the plates were incubated at room temperature for 1 hour with shaking, and then washed three times using 1× washing buffer. A warm substrate solution (100 μl) was added to each well, and the plates were then incubated for 20 minutes at room temperature. The enzyme reaction was stopped by adding 100 μl of stop solution to each well, and the absorbance at 450 nm was determined using a microplate reader.

### Measurement of hydroxyl radical scavenging activity

The hydroxyl radical scavenging activity of MCP, MCF and charantin was measured using a published method [15], with some modifications. Hydroxyl radical scavenging activity was assessed by monitoring the hydroxylation of salicylate by the Fe^3+^–salicylate–hydrogen peroxide (H_2_O_2_) system. The reaction mixture was composed of 1 ml of FeSO_4_ (1.5 mM), 0.7 ml of H_2_O_2_ (6 mM), 0.3 ml of sodium salicylate (20 mM) and 1 ml of the extracts (5–15 mg/ml), which were incubated for 1 hour at 37°C. Absorbance was measured at 562 nm. Mannitol was used as a positive control. The hydroxyl radical scavenging activity was calculated as:

$$Scavenging\;activity\;\left(\%\right)=\;1-\frac{\left(A_{1}-A_{2}\right)}{A_{0}}\times 100$$

where A_0_ was the absorbance of the control, A_1_ was the absorbance in the presence of the extract and A_2_ was the absorbance without sodium salicylate.

### Measurement of DPPH radical scavenging activity

DPPH radical scavenging activity of extracts was determined using an established procedure [16]. The extracts (15 mg/ml) of MCP, MCF and charantin were mixed with 0.5 ml of a methanolic solution containing DPPH (0.1 mM) and incubated for 30 min at 37°C in the dark. Absorbance was measured at 517 nm. Ascorbic acid was used as a positive control. Free radical scavenging activity was computed as

$$\frac{\left(A-B\right)}{B}\;\times 100$$

A = the absorbance of 0.5 ml of the extracts mixed with 0.5 ml of the DPPH solution.

B = the absorbance of 0.5 ml of the extracts mixed with 0.5 ml of methanol.

### Measurement of metal chelating activity

The ability of MCP, MCF and charantin extracts to chelate ferrous ions (Fe^2+^) was assessed using a modified version of an established procedure [17]. The reaction mixture was composed of 0.5 ml of the extract (5–15 mg/ml), 50 μl of FeCl_2_ (2 mM) and 0.6 ml of deionized water. The mixture was shaken and incubated at room temperature for 10 minutes. A volume of 100 μl of ferrozine (5 mM) was added, mixed and left for an additional 5 minutes to complex the residual Fe^2+^. The absorbance of the Fe^2+^–ferrozine complex was measured at 562 nm. EDTA was used as a positive control. The Fe^2+^-chelating activity of the extract was calculated using the following formula:

$$Chelating\;rate\;\left(\%\right)=[1-\frac{absorbance\;of\;sample}{absorbance\;of\;control}\;\times 100$$

### Measurement of reducing power

The reducing power of MCP, MCF and charantin was determined using an established procedure [18]. A 2.5 ml volume of the different extracts (15 mg/ml) was mixed with 2.5 ml of 0.2 M sodium phosphate buffer (pH 6.6) and 2.5 ml of 1% potassium ferricyanide (K_3_Fe(CN)_6_). The mixture was incubated for 30 minutes at 50°C. A 2.5 ml volume of 10% trichloroacetic acid (w/v) was added to all samples to stop the chemical reactions, which were then centrifuged at 1,000 rpm for 10 minutes. The supernatant (2.5 ml) was removed and added to 2.5 ml of distilled water and 0.5 ml of 0.1% FeCl_3,_ and the absorbance measured at 700 nm. Distilled water with no added extracts was used as a blank; ascorbic acid was used as a positive control.

### Measurement of total phenolic content

The amounts of the total phenolic compounds in MCP, MCF and charantin were estimated as described previously [19]. Aliquots of 1 ml of the ethanolic MCP, MCF and charantin extracts (10 g/l) were mixed with 5 ml of Folin–Ciocalteu reagent (diluted tenfold) and incubated for 3 minutes at room temperature. A 5 ml volume of a 10% Na_2_CO_3_ solution was added, and the mixture was incubated for 1 hour at room temperature. The method was calibrated using 0.24–0.30 mg/ml ethanolic gallic acid standards. Absorbance was measured at 760 nm, and the final results were expressed as mg/g of gallic acid equivalents.

### Measurement of total flavonol content

Flavonol content was determined using a colorimetric method [19]. Aliquots of 1 ml of the ethanolic MCP, MCF and charantin extracts (10 g/l) were mixed with 1 ml of AlCl_3_ in ethanol (20 g/l) and 3 ml of sodium acetate in ethanol (20 g/l); the mixture was incubated for 1 hour at 20°C. The standard curve was prepared using 0.0166–0.5 mg/ml rutin ethanolic solutions. Absorbance was measured at 440 nm and the flavonol content was expressed in milligrams of rutin per gram of extract.

### Measurement of total flavonoid content

Flavonoid content was determined using a previously described colorimetric method [19]. A 1 ml volume of the ethanolic MCP, MCF and charantin extracts (10 g/l) was mixed with equal volumes of 2% AlCl_3_ in ethanol (20 g/l). The mixture was shaken and incubated for 40 minutes at 20°C. Absorbance was measured at 415 nm, and flavonoid content was expressed as milligrams of rutin per gram of extract.

### Statistical analysis

Statistical analysis was performed using Microsoft Office Excel 2007. The results of each experiment are presentedas mean ± standard deviation (SD). The data were analyzed for significance using Student’s *t*-test or analysis of variance (ANOVA). Results with *p* values ≤ 0.05 were deemed statistically significant.

## Results

### Effects of MCP, MCF and charantin on AGEs

**Fig 1:**
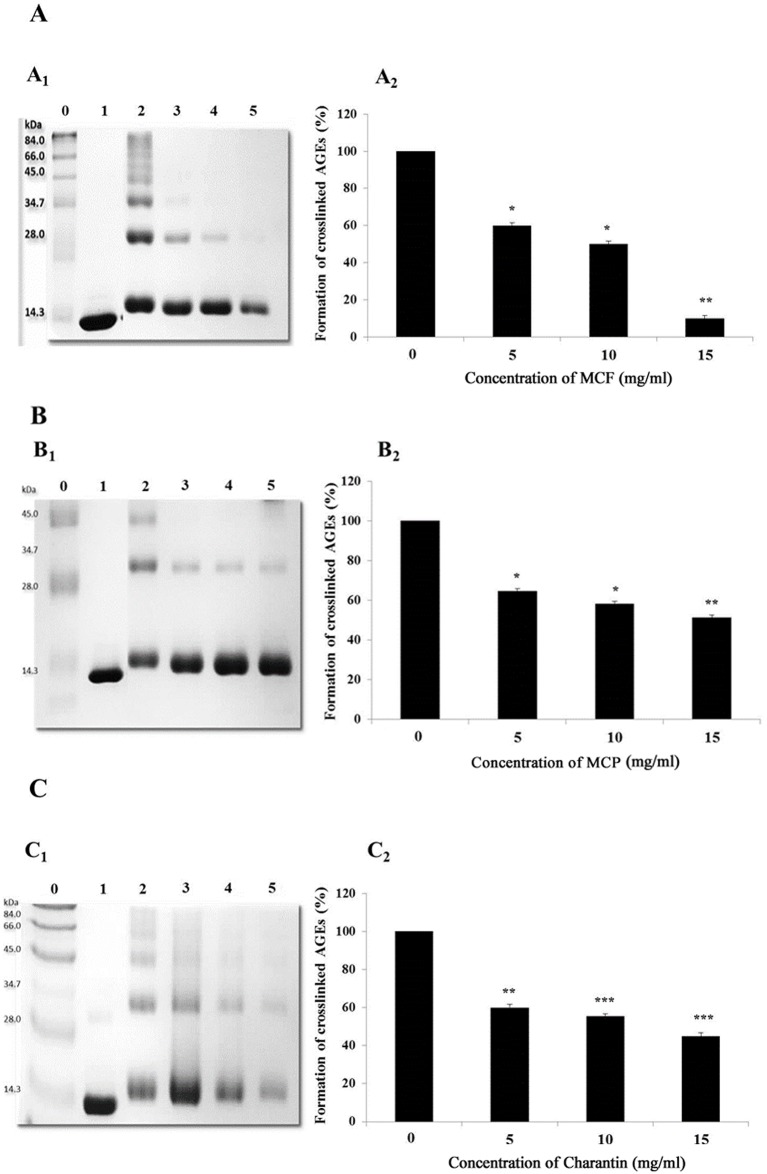
Effects of different concentrations of MCF (A), MCP (B) and charantin (C) on the formation of crosslinked AGEs. A representative SDS-PAGE gel showing lysozyme (10 mg/ml) incubated alone (lane 1) or in the presence of 0.1 M methylglyoxal (lane 2) in 0.1 M sodium phosphate buffer of pH 7.4 for 3 days at 37°C. The dimer formation resulting from protein crosslinking was determined using different concentrations of the MCF (A_1_) and MCP (B_1_) extracts or charantin (C_1_): 5 mg/ml (lane 3), 10 mg/ml (lane 4) and 15 mg/ml (lane 5). Lane 0 contains the marker proteins. The bar charts show the effects of different concentrations of MCF (A_2_), MCP (B_2_) and charantin (C_2_) on the formation of crosslinked AGEs relative to the control. The results are presented as means ± SDs (n = 3). *: *p* < 0.05, **: *p* < 0.01, ***: *p* < 0.001 vs control.

Methylglyoxal, a highly reactive endogenous metabolite, reacts with lysozyme at a higher rate than glucose (the major metabolic sugar), resulting in the formation of crosslinked AGEs within 3 days of incubation (Fig 1, lanes 2). The inclusion of MCP, MCF extracts and charantin inhibited the formation of methylgloyoxal derived crosslinked AGEs in a dose-dependent manner (Fig 1). Thus, dimerization of lysozyme during the glycation reaction was reduced in a dose-dependent manner with MCF extracts of 5, 10 and 15 mg/ml, resulting in 40% (*p* < 0.05), 55% (*p* < 0.05) and 88% (*p* < 0.01) inhibition of the formation of crosslinked AGEs, respectively, compared with the control (Fig 1A). The formation of crosslinked AGEs was also significantly inhibited when lysozyme was glycated in the presence of the MCP extract, compared with the control. This inhibition depended on the concentration of MCP used, with the greatest inhibition (49%, *p* < 0.001) occurring when a 15 mg/ml MCP extract was used (Fig 1B). Similarly, charantin exhibited the greatest inhibition (55%, *p* < 0.001) when it was used at a concentration of 15 mg/ml (Fig 1C). Overall, the MCF extract was a more effective inhibitor of AGE formation than MCP or charantin (Fig 1).

**Fig 2:**
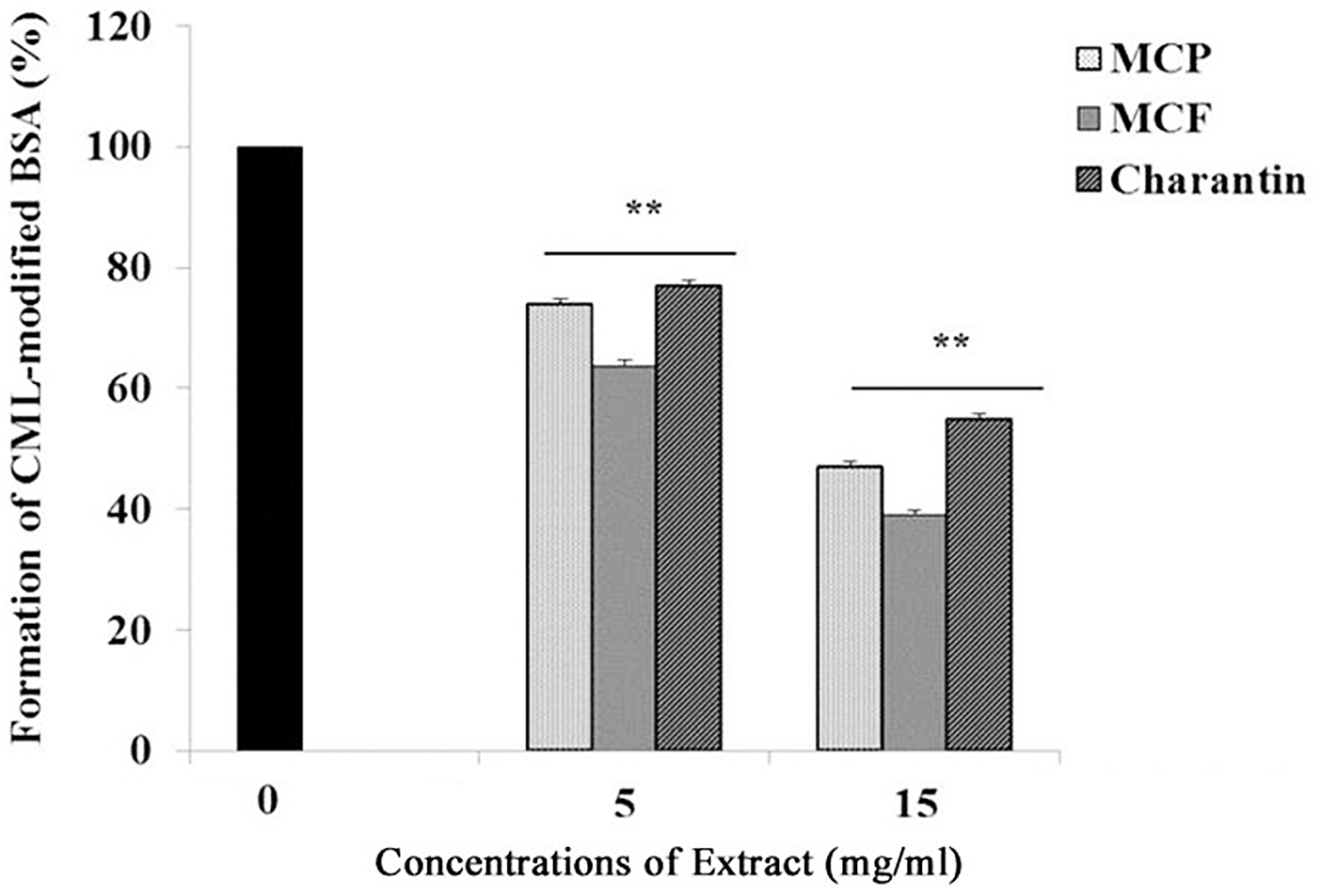
Effects of different concentrations of MCP, MCF and charantin on CML formation. The bar graph shows the effect of different concentrations (5 and 15 mg/ml) of the MCP, MCF and charantin extracts on production of CML following glycoxidation of BSA (300 mg) in 0.2 M phosphate buffer, pH 7.8 at 37°C over a period of 24-hours. The positive control was the amount of CML-modified BSA produced in the absence of extracts. The results are presented as mean ± SD (n = 3), **: *p* < 0.01 vs control.

In addition to the evaluation of crosslinked AGE formation, the effect of *M*. *charantia* extracts on the formation of CML (an oxidative AGE) was also investigated. The positive control was the amount of CML-modified BSA that was produced by glyoxylic acid. Compared to the positive control, all the extracts inhibited CML formation, with the strongest inhibition exhibited by the MCF extract (Fig 2). Moreover, the inhibition depended on the concentration of the extract used. At concentrations of 5 and 15 mg/ml, MCF significantly (*p* < 0.01) inhibited CML formation by 37% and 61%, respectively. Also at concentrations of 5 and 15 mg/ml, MCP significantly (*p* < 0.01) reduced CML formation by 26% and 53%, respectively. Furthermore, charantin at 5 and 15 mg/ml concentrations significantly (*p* < 0.01) decreased CML formation by 23% and 45%, respectively (Fig 2).

### Antioxidant properties of MCP, MCF and charantin

**Fig 3:**
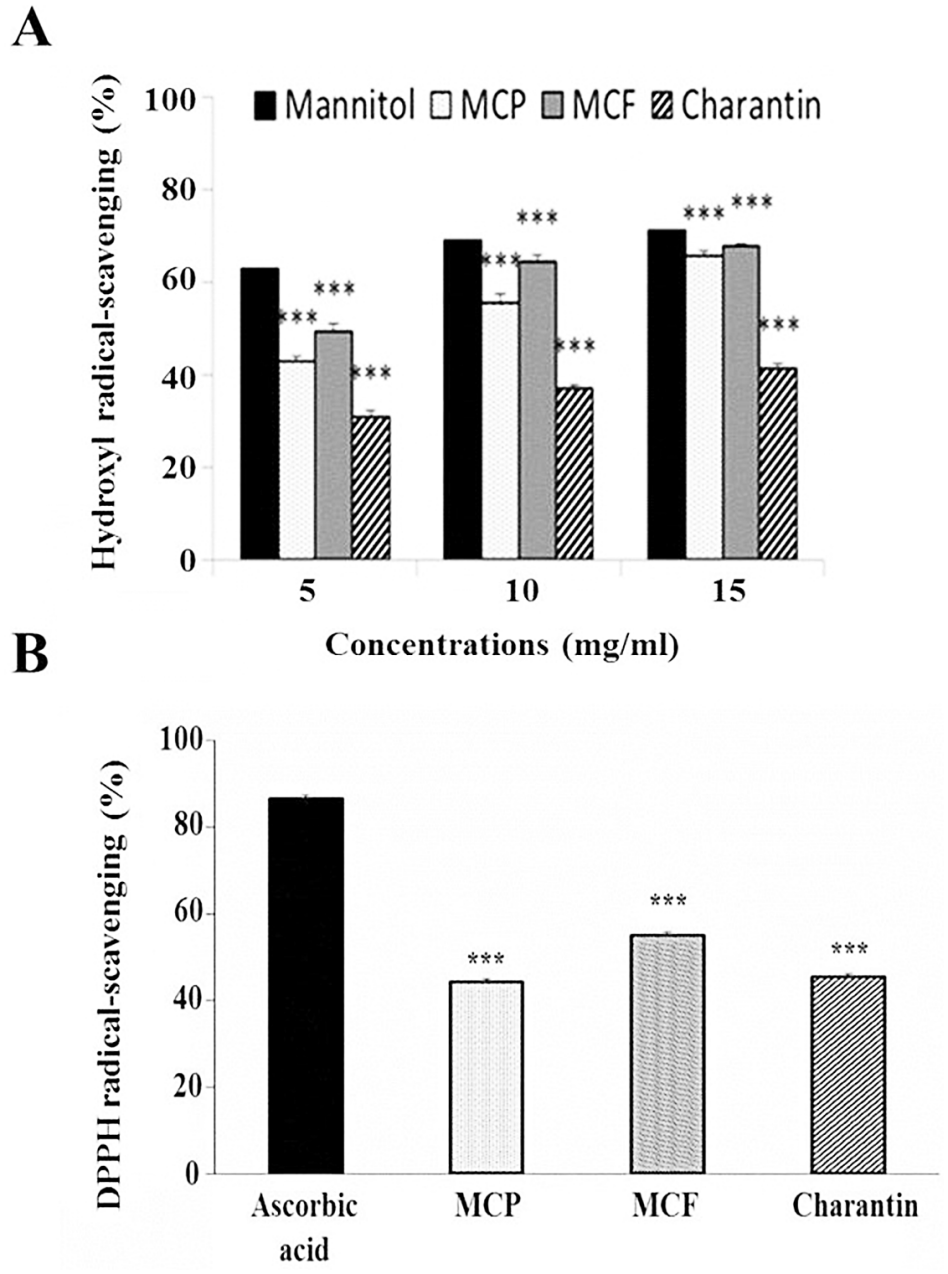
Radical scavenging activity of different concentrations of MCP, MCF and charantin. (A) The hydroxyl radical scavenging activity of 5–15 mg/ml extracts of MCP, MCF and charantin was assessed by monitoring the hydroxylation of salicylate by the Fe^3+^-salicylate-H_2_O_2_ system over a period of 30 minutes at 37°C. Mannitol was used as a positive control. (B) The DPPH radical scavenging activity of 15 mg/ml extracts of MCP, MCF and charantin at 37°C over a period of 30 minutes was also assessed. Ascorbic acid was used as a positive control. All results are presented as mean ± SD (n = 3). ***: *p* <0.001 vs control.

The hydroxyl radical scavenging abilities of the MCP, MCF and charantin extracts were determined and compared (Fig 3A). The MCP, MCF and charantin extracts increased hydroxyl radical scavenging activity in a dose-dependent manner (Fig 3A). The MCF extract had a hydroxyl radical scavenging activity of 70% (*p* < 0.001) when used at 15 mg/ml whereas that for MCP extract was 68% (p < 0.001) at the same concentration. Charantin at the same concentration of 15 mg/ml had the lowest hydroxyl radical scavenging activity of 45% (*p* < 0.001). The hydroxyl radical scavenging activities of MCP, MCF and charantin were less than that of mannitol, the latter being used as a positive control with 75% scavenging activity at 15 mg/ml (Fig 3A).

The effects of MCP, MCF and charantin on DPPH radical-scavenging activity are shown in Fig 3B. The percentage inhibition of free radical activity was used to evaluate the protective effects of a 15 mg/ml solution of the MCP, MCF and charantin extracts on DPPH radical scavenging activity *in vitro* (Fig 3B). The DPPH radical scavenging activities of MCP, MCF and charantin extracts were significantly lower than that for the positive control ascorbic acid (*p*< 0.001; Fig 3B) which showed approximately 85% scavenging of DPPH radicals. The scavenging activity of the MCF extract was 55% and higher than that of the MCP (40%) or charantin (45%) extracts (Fig 3B).

**Fig 4:**
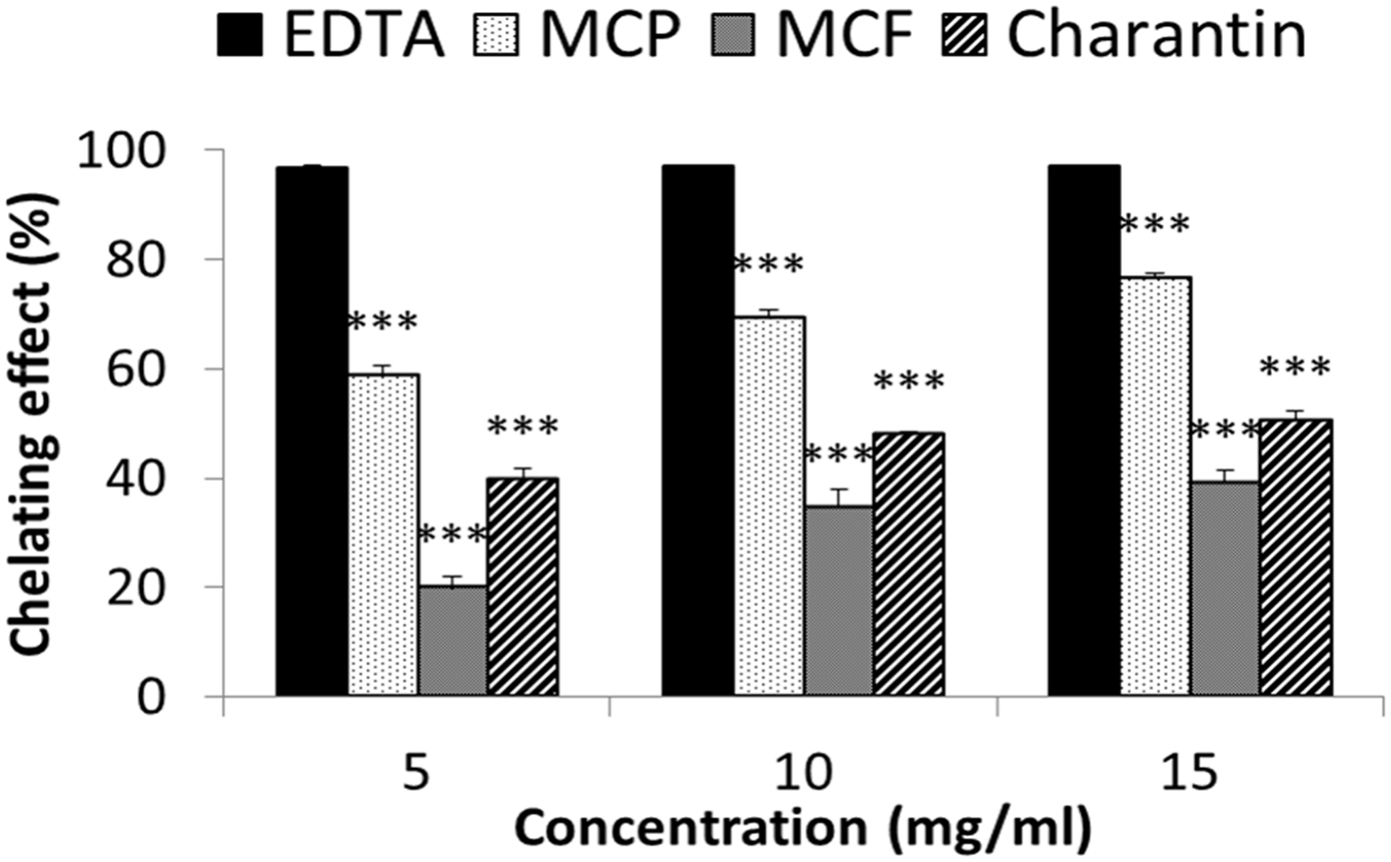
Metal chelating activity of different concentrations of MCP, MCF and charantin. Chelating effects of 5–15 mg/ml of MCP, MCF and charantin extracts were determined over a period of 10 minutes at room temperature using the ferrozine method. EDTA was used as a positive control. The results are presented as mean ± SD (n = 3). ***: *p* <0.001 vs control.

**Fig 5:**
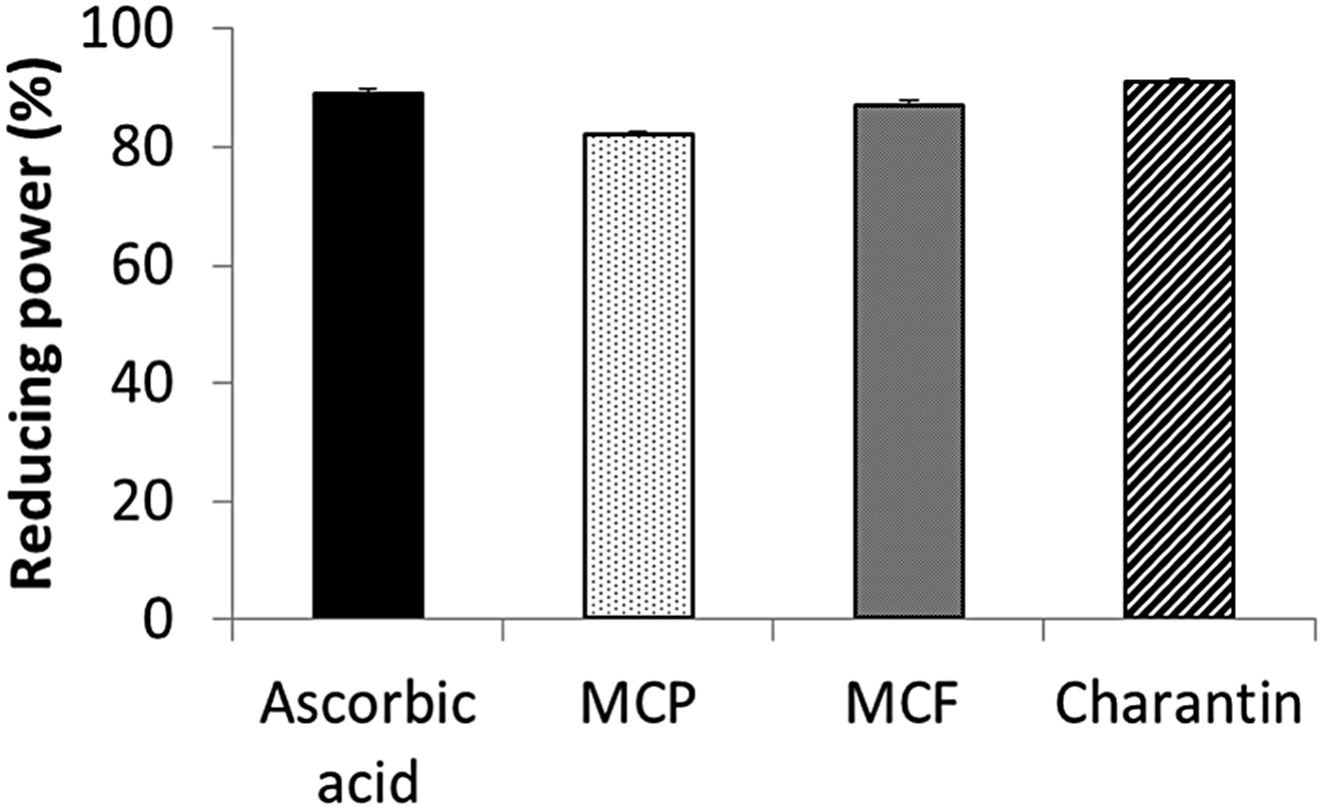
The reducing power of 15 mg/ml extracts of MCP, MCF and charantin. Reducing power of MCP, MCF and charantin (15 mg/ml) extracts were determined over a period of 30 minutes at 50°C using the potassium ferricyanide method. Ascorbic acid was used as a positive control. The results are presented as mean ± SD (n = 3). There was no significant difference between the extracts and ascorbic acid.

MCP, MCF and charantin also increased metal-chelation in a dose-dependent manner (Fig 4). The highest chelating effect was observed at a concentration of 15, then 10 and finally 5 mg/ml for all three extracts. Of the three, MCP was the most effective with 80% chelation at a concentration of 15 mg/ml compared to the 40% for MCF and 30% for charantin at the same concentration (Fig 4). EDTA as the positive control had the most potent chelating activity of approximately 100% at all three concentrations used. Indeed at all concentrations used the chelating effects of extracts were significantly lower than that for EDTA (p<0.001). Furthermore, MCP, MCF and charantin extracts showed significant reducing power, as demonstrated by their ability to reduce ferric ions to ferrous ions. Indeed, the reducing power of MCP, MCF and charantin extracts were 83%, 87% and 90% respectively compared to 89% for ascorbic acid the positive control (Fig 5). There was no statistical difference between the reducing power of the three extracts and the ascorbic acid. Thus at the concentrations used, all three extracts had effective reducing power similar to ascorbic acid.

**Fig 6:**
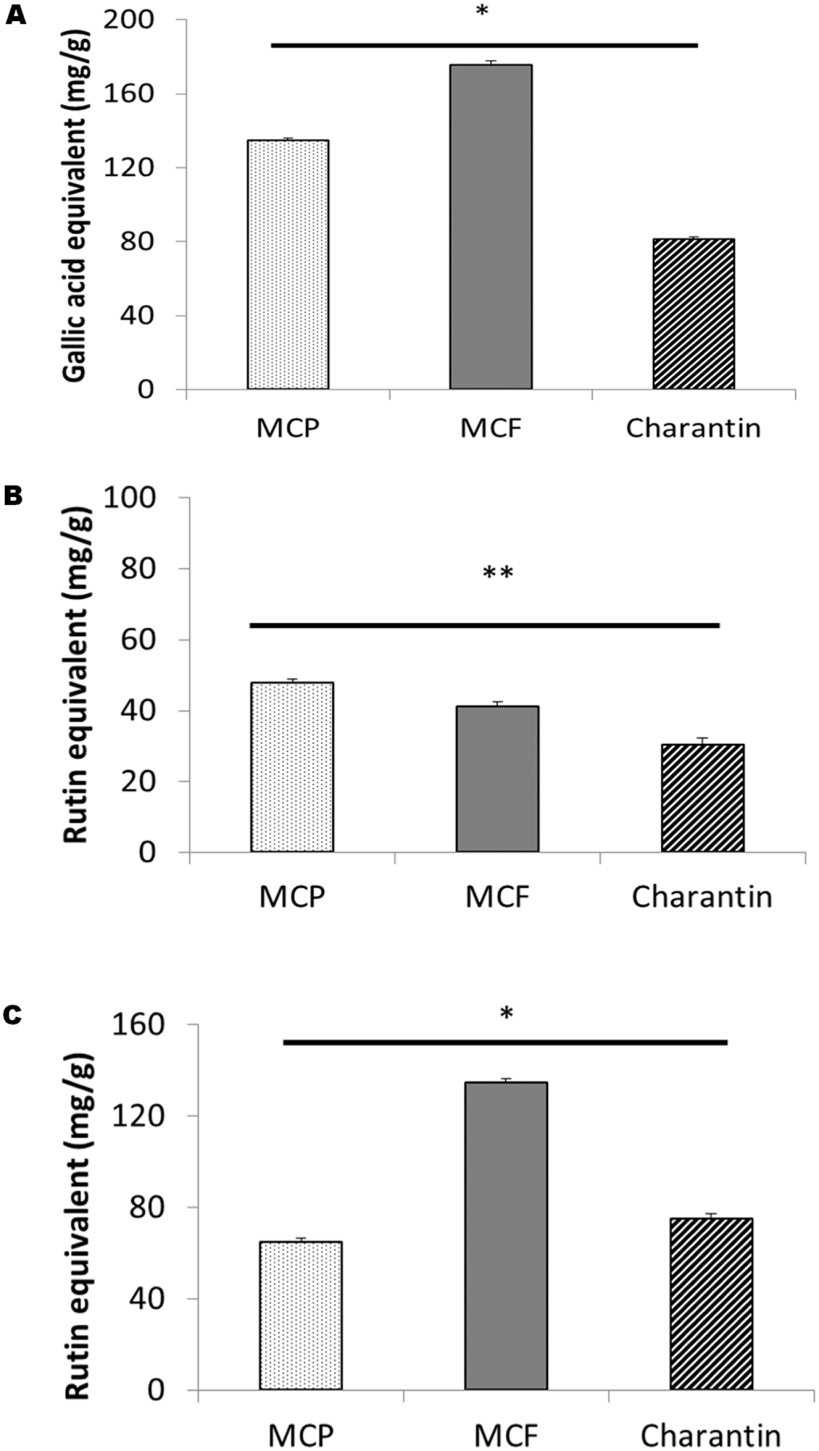
Total phenolic, flavonol and flavonoid contents of 10 mg/ml solutions of MCP, MCF and charantin. (A) phenolic content was determined using the Folin-Ciocalteau method calibrated using gallic acid standards with the results expressed as mg/g of gallic acid equivalents, (B) flavonol content was determined using the AlCl_3_ method where the method was calibrated using rutin and results expressed as mg of rutin per g of extract and (C) flavonoid contents of the ethanolic MCP, MCF and charantin extracts determined by a colorimetric method calibrated using rutin and the results expressed as mg of rutin per g of extract. The results are presented as mean ± SD (n = 3). *: p < 0.05, **: *p* < 0.01.

The phenolic content of the extracts differed significantly (Fig 6A). The MCF extract had a significantly higher (*p* < 0.05) phenolic content (175.33 ± 2.30 mg/g of gallic acid equivalents) than that of the MCP extract (134.66 ± 1.15 mg/g) and the charentin extract (81 ± 1.51 mg/g; Fig 6A). However, in contrast the highest flavonol content was observed in the MCP extract, with a value of 47.86 ± 0.91 mg/g of rutin equivalent, whereas the values for the MCF extract and charantin were 41.11 ± 1.38 mg/g and 30.33 ± 1.90 mg/g, respectively (Fig 6B). The flavonoid contents ranged from as high as 134.67 ± 1.89 mg/g of rutin equivalent in the MCF extract to as low as 64.55 ± 2.03 mg/g in the MCP extract (Fig 6C). Indeed, the total flavonoid content of the MCF extract was significantly higher (*p* < 0.05) than that of the MCP and charantin extracts (Fig 6C).

## Discussion

Protein glycation results in the accumulation of AGEs, which cause protein modifications that result in structural and functional impairments and the formation of intra- and inter-protein crosslinking [20]. The accumulation of crosslinked AGEs in tissues is believed to be one factor responsible for the long-term complications of diabetes and aging [3]. Studies have shown that a range of medicinal plants can reduce AGE formation *in vitro*, which suggests that such plants could potentially provide protection against the development of such complications [20, 21]. One of the best studied medicinal plants with antidiabetic effects is *M*. *charantia* [10], a plant with known hypoglycaemic properties in both animals [22] and humans.

In the present study, the antiglycation and antioxidant properties of MCP, MCF and charantin extracts from *M*. *charantia* were studied *in vitro*. The experiments involved *in vitro* protein glycation by incubation with methylglyoxal at physiological pH, although a non-physiological concentration of methylglyoxal was used to accelerate AGE formation over a shorter time period. Lysozyme was used because it is a good model protein for the measurement of crosslinked AGEs, as oligomerization occurs readily and is easily measurable by SDS-PAGE. The glycation of lysozyme *in vitro* has been examined in several previous studies [23]. In this study, MCP and MCF extracts and charantin showed potent dose-dependent inhibitory effects on methylglyoxal-induced AGE formation *in vitro*. The ability of certain plant extracts to inhibit the formation of AGEs is thought to be related to their antioxidant properties, and particularly their ability to scavenge radicals formed during the glycation reaction [24, 25].

We showed that *M*. *charantia* extracts have antioxidant effects (in that they inhibited free radical activity), and that they also possessed antiglycation potential. Indeed, this protection against glycation-induced crosslinking may be related to the ability of the extracts to chelate transition metals, which was shown in the current study and could potentially prevent autoxidative glycation and glycoxidation reactions. In a previous study, *M*. *charantia* extracts were shown to chelate Cu^2+^ ions and protect against low-density lipoprotein oxidation [26].

MCF had a more potent inhibitory effect on the formation of AGEs compared with MCP and charantin, which might be attributed to its higher phenolic and flavonoid content. Several studies have demonstrated that *M*. *charantia* contains numerous amino acids that might reduce AGE formation by blocking the carbonyl groups on glycated proteins [27]. CML is among the most abundant AGE produced *in vivo* from glycation and oxidation reactions [28]. Elevated CML levels have been identified in diabetic animal and human tissues [29, 30]. The current study confirms that all three *M*. *charantia* extracts can inhibit CML formation *in vitro*, with MCF having the most potent effect. Several studies have shown that *M*. *charantia* significantly lowers blood glucose concentrations in diabetic patients [31] and in diabetic rats [10]. These study findings indicate that *M*. *charantia* extracts might reduce AGE formation by lowering glucose levels.

Low-molecular-weight antioxidant compounds, such as polyphenols, flavonoids and flavonols, have been identified in *M*. *charantia* [25, 32]. The results of the present study show that MCF extract, which contains a significantly higher phenolic content than MCP or charantin, shows the greatest antioxidant activity. Polyphenolic compounds might be responsible for the free radical scavenging activities associated with *M*. *charantia* extracts. A report has suggested a relationship between the free radical scavenging activity of polyphenolic substances and their chemical structures [33]. Other amine-group-containing molecules may be present in the extract that are capable of reacting with methylglyoxal, thus protecting the amino groups in proteins from being glycated. Indeed, other antioxidant molecules may be present in the extract that can protect against autoxidative glycation, thus reducing the formation of AGEs. Additionally, the possibility of synergistic effects between different extract constituents cannot be ignored and are often viewed as an advantage of using extracts.

This study investigated the theory that phenolic compounds in certain plant extracts effectively inhibit the formation of AGEs. Thus, phenolic substances can play an important role in antiglycation and antioxidant activities. All extracts in the current study inhibited the formation of AGEs, with MCF being the most potent. In addition, all extracts had antioxidant properties and reduced oxidative stress. The metal chelating activity of these extracts probably has a major role in their ability to inhibit AGE formation [34].

A pilot study has been conducted involving a double-blinded, placebo controlled trial in which type 2 diabetic patients were given either 6g/day of *M*.*charantia* pulp or placebo for 16-weeks [35]. After this period, there was a significant reduction of both glycated haemoglobin (HbA_1c_) and serum fluorescent AGEs [35]. However, it is known that *M*.*charantia* has hypoglycaemic properties thus the reduction in serum AGEs might be due to better glycaemic control rather than a direct effect of the extract on AGE formation. Furthermore, this study did not measure markers of oxidative stress. Our studies albeit *in vitro* demonstrates that *M*.*charantia* extracts have a direct protective effect against AGE formation and oxidative stress. While this manuscript was being prepared, another study also demonstrated the antiglycation properties of *M*. *charantia* extracts, thus confirming our studies [36]. Although some evidence exists that the consumption of *M*. *charantia* can reduce hyperglycaemia, it is not clear whether there is a direct effect on reduction of AGEs *in vivo*. However, *M*.*charantia* deserves more attention as a low-cost food for diabetes management with the possibility of protection against diabetes-induced complications.

## Abbreviations

AGE: advanced glycation endproduct
BSA: bovine serum albumin
CML: carboxymethyllysine
DPPH: 1,1-diphenyl-2-picryl-hydrazyl
EDTA: ethylenediaminetetraacetic acid
ELISA: enzyme-linked immunosorbent assay
I.D.: integrated density
MCF: *Momordica charantia* flesh
MCP: *Momordica charantia* pulp
SD: standard deviation
SDS-PAGE: sodium dodecyl sulfate-polyacrylamide gel electrophoresis
